# Influence of oxygen deficiency and the role of specific amino acids in cryopreservation of garlic shoot tips

**DOI:** 10.1186/s12896-015-0171-7

**Published:** 2015-05-28

**Authors:** Karthikeyan Subbarayan, Hardy Rolletschek, Angelika Senula, Kamatchi Ulagappan, Mohammad-Reza Hajirezaei, E.R. Joachim Keller

**Affiliations:** Leibniz Institute of Plant Genetics and Crop Plant Research (IPK), Corrensstr. 3, D-06466 Gatersleben, Germany

**Keywords:** *Allium sativum*, PVS3, Hypoxia, Cryo-stress

## Abstract

**Background:**

Garlic has lost its ability to form seeds in the course of its domestication. Therefore, the germplasm storage via cryopreservation is increasingly applied. The progression of the various steps within the cryopreservation procedure is accompanied by declining survival rates of the explants. Much of the recent work on cryo-stress has been focussed on osmotic and cold stress components. However, two decades after invention of garlic cryopreservation, the function of metabolites and oxygen in and around the cryopreserved tissues is still largely obscure.

**Methods:**

In this study, hypoxia was characterized in cryopreservation of garlic with oxygen sensors and amino acid metabolism. Furthermore, malondialdehyde, soluble sugars and ammonium were quantified to demonstrate the influence of cryo-stress in declining survival rates.

**Results:**

To better understand the possible reasons for a reduction in the survival rate at the subsequent steps of cryopreservation, the concentration of amino acids, ammonium, γ-aminobutyric acid (GABA), soluble sugars, malondialdehyde (MDA), and oxygen were measured in garlic shoot tips undergoing cryopreservation. Using microsensors, a very low oxygen concentration (<0.1 μM) was detected within the central meristem region of the shoot apex. When apices were immersed in cryoprotectant solution, the well-oxygenated peripheral regions (foliage leaf bases) became likewise hypoxic within a few minutes, probably resulting from strongly restricted gaseous diffusion.

**Conclusions:**

Tissue level oxygen measurements supported the occurrence of hypoxia while biochemical analysis indicated adaptive responses, in particular the modulation in alanine and glutamate metabolism. The possible role of serine and glycine metabolism during cryopreservation is also discussed.

## Background

Cryopreservation, the storage of germplasm at ultra-low temperature in liquid nitrogen, was successfully applied to many plant species [1, 2]. It is considered as an important tool also for long-term conservation of garlic. Shoot apices are the preferred tissue source for garlic cryopreservation [3]. Meristem cells located in the apical region of shoot tips have higher tolerance towards cryo-storage protocols [4]. Among several cryopreservation protocols, vitrification is characterized by initiating glass transition of the cellular water content. This avoids ice-caused damage during freezing. The plant vitrification solution PVS3 [5], is the most effective for garlic allowing higher regrowth rates than other vitrification solutions [6]. The genebank at IPK comprises one of the largest cryo-collections worldwide with more than 1500 accessions, the number of garlic accessions amounting to 106. In general, regrowth of plants after cryopreservation varies, and resulting rates range from poor to excellent. Understanding how and why germplasm does or does not survive cooling is needed to further improve plant cryopreservation [1]. In nature, plants are not exposed to such low temperatures as during freezing. Cryogenically-stored plant tissues undergo dehydration, osmotic, oxidative and cold stress conditions in the course of the cryopreservation procedure [7]. While lipid peroxidation was well documented as occurring under cryo-stress by measuring malondialdehyde (MDA) accumulation [8], none of these studies has discussed the potential role of the amino acid metabolism under the specific physiological conditions occurring during cryopreservation. Furthermore, the oxygen level in the cryopreservation solutions as well as in the tissue exposed to dehydration solution was not yet recorded, while the osmotic effects were demonstrated [9]. Within a plant tissue or organ, cells are frequently challenged with limited levels of oxygen supply due to changes in the external environment or high rates of cellular metabolism [10, 11]. This in turn causes partial or complete inhibition of mitochondrial respiration, also known as hypoxia or anoxia, respectively.

To gain insights into deleterious effects of cryo-stress, we investigated the amino acid content in the tissue in the course of the cryopreservation procedure. We found an altered modulation in alanine and glutamate metabolism at dehydration step that may indicate hypoxic stress in the tissue [12]. Using microsensors, O_2_ maps were generated for garlic shoot apices providing evidence for severe O_2_ deficiency in shoot apices. We demonstrated that some of the metabolites are specifically regulated during dehydration and after storage in liquid nitrogen (LN). Furthermore, accumulation of MDA was confirmed to play an important role in cryo-stress. On the basis of our results, the role of amino acid metabolism in response to hypoxic stress in cryopreserved garlic shoot apices is discussed.

## Methods

### Plant material

**Fig. 1:**
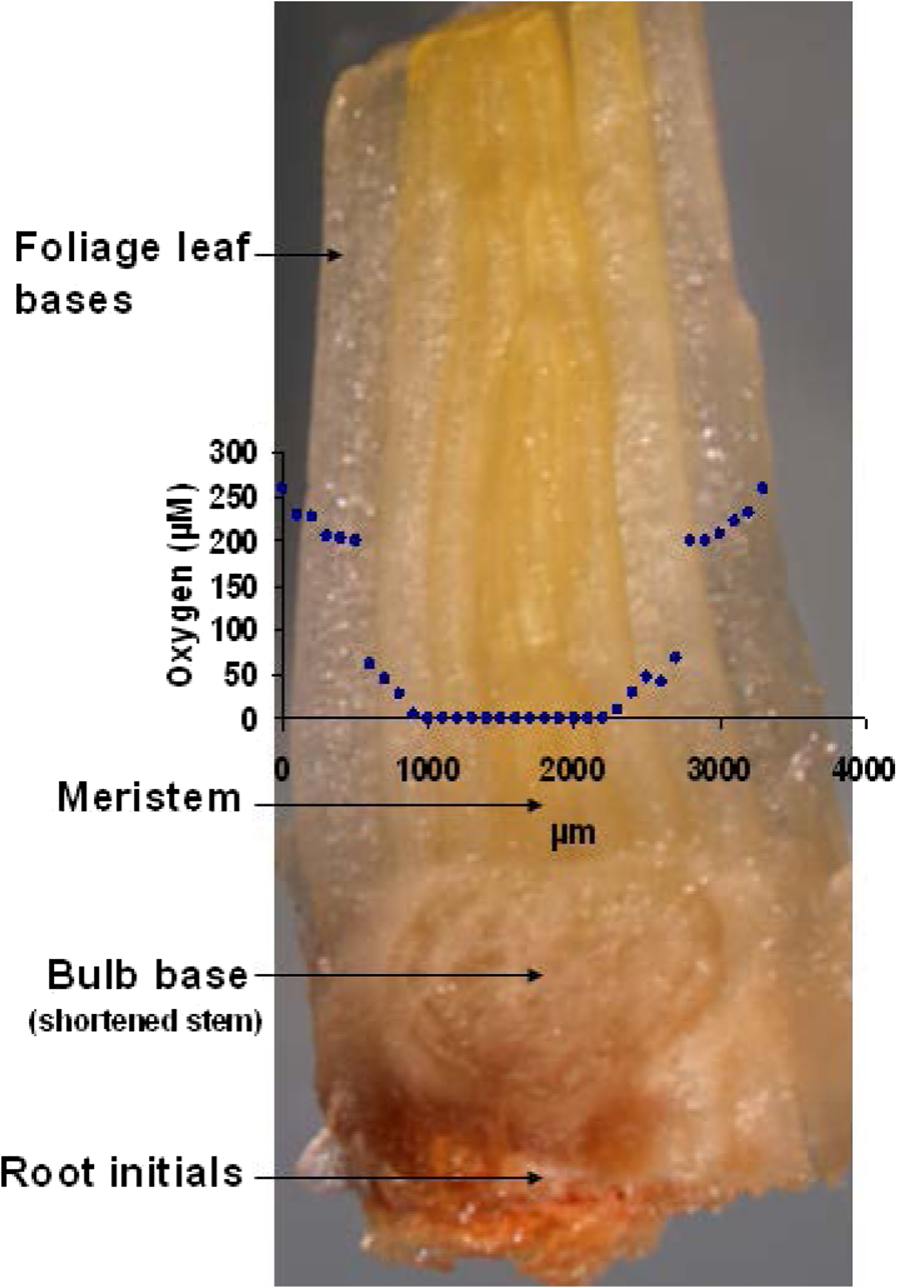
O_2_ map of shoot apices of garlic: Structural relations (longitudinal cut) and O_2_ gradient. Using a microsensor, O_2_ was measured along the x-axis (penetration depth given in μm). The O_2_ concentration is given in μM (254 μM corresponds to atmospheric saturation at 25 °C)

Bulbils of garlic (accessions All0292, All0841, All1166, and All1171,) were obtained from field grown plants of the genebank at IPK. In 2012 and 2013, summer-harvested bulbils were stored under the wooden roof until the end of winter. In vitro cultures were maintained via cyclic micropropagation using standard medium consisting of MS [13] with 0.1 mg/L naphthalene acetic acid (NAA), 0.5 mg/L N6-(2-isopentenyl) adenine (2iP), 30 g/L sucrose, and 10 g/L agar, pH 5.8 [14, 3] and maintained at 25 °C and 16 h illumination of 60 μmol m^−2^ s^−1^. Light-microscopic images of shoot apices display the meristem with two or three surrounding foliage leaf bases (Fig. 1). The base of shoot apices is embedded into the bulbils or in vitro plantlets on the center of the basal plate.

### Explant sterilization

Bulbils were treated with 70 % ethanol for 10–20 s, and then shaken in sodium hypochlorite solution (3 % active chlorine [Carl Roth GmbH & Co., Karlsruhe, Germany]) with three drops of Tween 20 [Riedel-de Haën AG, Seelze, Germany] for 25 min. Bulbils were then rinsed five times with sterile water. Shoot apices 1–2 mm in diameter and 3 mm long were excised from bulbils [3].

### Cryogenic treatment procedure

The cryopreservation procedure was followed as described by Keller and Senula [3]. The procedure consisted of four steps. In all experiments, generally 20 shoot tips were used per experimental step. Experiments were replicated 2–4 times. The steps were denominated by letters which was also used in the graphs:

F Fresh: Shoot apices were excised from garlic bulbils or in vitro plantlets.
P Preculture: Thereafter, the shoot apices were precultured in standard medium containing 10 % sucrose overnight.
D Dehydration: A dehydration step followed, in which the explant was treated with loading solution (18.4 % w/v glycerol and 13.7 % w/v sucrose in liquid MS medium) for 20 min and then dehydrated using PVS3 (50 % w/v glycerol and 50 % w/v sucrose in liquid MS) for 120 min at 25 °C.
LN Storage and recovery (LN storage): Finally, the vitrified explants were rapidly cooled down and kept in liquid nitrogen for 60 min, then thawed at 40 °C in a water bath for 2 min, subsequently immersed in washing solution (1.2 M sucrose in liquid MS medium) for 10 min at 25 °C and finally plated onto solid MS medium.

### Determination of survival rates

Shoot apices from each of the above cryopreservation steps were propagated on standard medium for fifteen days and tested for survival rates by counting shoot apices that exhibited visible growth (F and P) or pertained greenish and swollen (D and LN). The data were calculated to display the results in percent of survival ± standard error.

### Determination of oxygen concentration in plant tissue and cryopreservation solutions using an optical microsensor

Oxygen concentration profiles across the tissue were measured using a needle-type microsensor according to Rolletschek *et al.* [15] at ambient atmosphere and incubation in cryopreservation solutions (vitrification, loading and washing solutions). Briefly, an oxygen-sensitive optodes (Presens GmbH, Regensburg, Germany) was inserted and moved forward into the tissue using a micromanipulator, and the oxygen concentration was measured at 100 μm intervals along the horizontal pathway of sensor penetration. Before and after each analysis the sensor was calibrated using premixed gases (0 and 21 kPa oxygen, balanced by N_2_). Measurement of oxygen concentration in cryopreservation solutions was done by inserting the microsensor into the gently stirred solution for at least 1 min.

### Fresh and dry weight comparison

The explants were harvested after each step of the cryopreservation procedure and fresh as well as dry weight were measured. For dry weight determination explants were dried in a mechanically ventilated oven at 100 ± 2 °C for 24 h to determine dry weight (DW). As fresh weight is influenced by the reduction of water content due to progressive dehydration during cryopreservation, percentage of DW was substituted in place of FW. DW % was calculated as follows

$$ \mathrm{D}\mathrm{W}\ \% = \left(\mathrm{D}\mathrm{W}/\mathrm{F}\mathrm{W}\right)*100 $$

### Quantification of malondialdehyde (MDA) by HPLC

Four shoot apices (40–80 mg fresh weight) were collected from each step of the cryopreservation procedure. MDA was measured according to Lepage *et al.* [16] with a few modifications. Shoot apices were added to 1 ml of 5 % TCA solution (trichloroacetic acid) and 0.1 ml of 20 mM BHT (butylated hydroxytoluene) and incubated at 95 °C for 30 min. After centrifugation at 1000 g for 10 min, an equal volume of 0.25 % thiobarbituric acid (TBA) was added to the supernatant. The reaction mixture was heated at 95 °C for 30 min for the formation of MDA-TBA complex. Aliquots of samples were transferred into microvials, which were placed in an autosampler and were automatically injected into the HPLC reverse-phase system (Hypersil ODS C_18_ with 5 μm particle size, 4.6 × 100 mm). The MDA (TBA)_2_ adduct was eluted using an isocratic mobile phase consisting of 100 % methanol at a flow rate of 0.2 ml/min at 25 °C. MDA was detected by fluorescence at excitation 532 nm and emission 553 nm. Quantification was done using an external standard of 1,1,3,3-tetraethoxypropane (Sigma, St Louis, MO) prepared using the same method as for the samples. Chromatograms were analyzed by EMPOWER (Waters, Germany).

### Determination of soluble sugars

Three shoot apices (30–40 mg fresh weight) were collected from each step of the cryopreservation procedure. The samples were extracted with 80 % ethanol at 60 °C and centrifuged at 14,000 g for 15 min. Soluble sugars (glucose, fructose and sucrose) were measured using an enzyme-coupled assay according to Hajirezaei *et al.* [17].

### Extraction and determination of free amino acids and ammonium

Plant material (30–40 mg fresh weight) was incubated for 60 min at 80 °C in 0.5 ml of 80 % ethanol and thereafter centrifuged for 10 min at 14,000 rpm and 4 °C. Supernatant was evaporated to dryness, re-suspended in purest water and analysed by ultra performance liquid chromatography (UPLC). Prior to UPLC analysis samples were derivatized using the fluorescing reagent AQC (6-aminoquinolyl-N-hydroxysuccinimidylcarbamat). 3 mg of self-made AQC (IPK, Germany) was dissolved in 1 ml acetonitrile and incubated exactly for 10 min at 55 °C. The prepared reagent was stored at 4 °C and used up within four weeks. For derivatization of the sample 0.01 ml of the prepared reagent solution was used for each sample which contained 0.8 ml of a buffer (0.2 M, pH 8.8) and 0.01 ml of the supernatant. Separation of soluble amino acids was achieved by a newly developed UPLC-based method using ultra pressure reversed phase chromatography (AcQuity H-Class, Waters GmbH, Germany). The UPLC system consisted of a quaternary solvent manager, a sample manager-FTN, a column manager and a fluorescent detector (PDA eλ Detector). The separation was carried out on a C18 reversed phase column (ACCQ Tag Ultra C18, 1.7 μm, 2.1x100 mm) with a flow rate of 0.7 ml/min and duration of 10.2 min. The column was heated at 50 °C during the whole run. The detection wavelengths were 266 nm for excitation and 473 nm for emission. The gradient was accomplished with four solutions prepared from two different buffers purchased from Waters GmbH (eluent A concentrate and eluent B for amino acid analysis, Waters GmbH, Germany and LCMS water, Geyer GmbH, Germany). Eluent A was pure concentrate, eluent B was a mixture of 90 % LCMS water and 10 % eluent B concentrate, eluent C was pure concentrate of eluent B and eluent D was LCMS water. The column was equilibrated with eluent A (10 %) and eluent C (90 %) for at least 30 min.

## Results

### Responses of shoot apices to cryogenic protocol

**Fig. 2:**
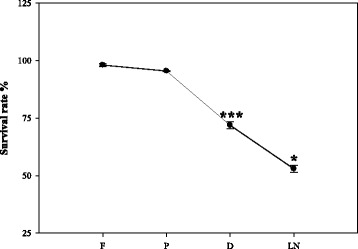
Survival rates after the consecutive steps of the cryopreservation procedure (F- fresh; P – preculture; D – dehydration; LN – Storage and recovery). The data represent the mean ± SE of 3 measurements. *p < 0.05. ***p < 0.001 using a paired Student’s t test

The response of shoot apices after each step of the PVS3 vitrification protocol was evaluated. Fresh explants showed the highest survival rates (98 – 100 %), which decreased (*P* < 0.05, paired Student’s *t*-test) after each step of the cryopreservation procedure (dehydration and LN storage) (Fig. 2). Finally after accomplished cryopreservation of garlic (and recovery (step LN) a survival rate of 47.1 % was determined.

### Oxygen mapping across shoot apices of garlic

Fine fiber optic microsensors (tip diameter ~ 30 μm) were used to measure O_2_ concentration across fresh shoot apices. The microsensors penetrated parallel to the bulb base targeting the meristem. The O_2_ level immediately declined to 20 % atmospheric saturation (~50 μM or 4 kPa oxygen) when the microsensor penetrated the foliage leaf bases, whereas a sharp decrease was detected at meristem region (Fig. 1). Within the central meristematic zone the oxygen concentration reached almost zero (<0.1 μM), indicating strong hypoxic conditions.

### Submergence of shoot apices in cryopreservation solutions causes significant reductions in endogenous oxygen levels

**Fig. 3:**
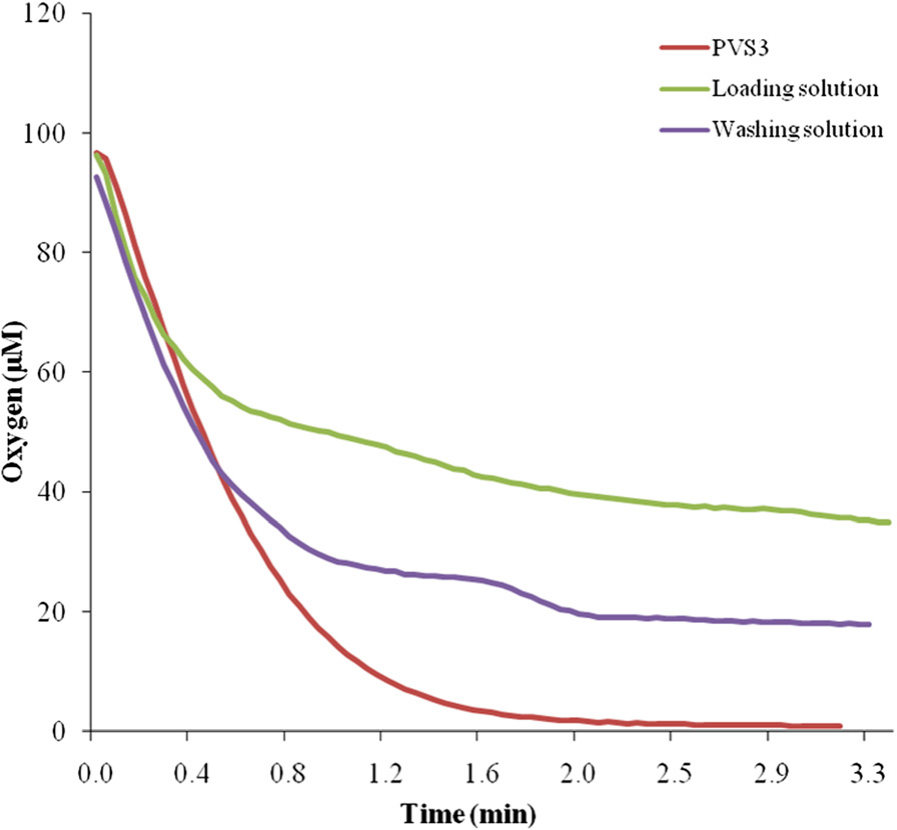
Dynamics of oxygen level in foliage leaf bases declining in response to the immersion in cryopreservation solutions. In PVS3, the maximum reduction of oxygen was observed followed by washing and loading solutions

Using microsensors, the oxygen level was measured in the solutions used for the cryo-procedure. The lowest O_2_ level was determined in PVS3 (70.8 ± 0.7 μM), followed by the washing solution (241.0 ± 1.2 μM), and loading solution (281.9 ± 0.4 μM). To monitor the interior of shoot apices during incubation in cryopreservation solutions, the microsensor was inserted into the shoot apex (~300 μm), where O_2_ levels were approx. 100 μM. The endogenous O_2_ level declined instantly upon immersion of shoot apices with either loading solution, PVS3 or washing solution (Fig. 3). The most severe decline was observed using PVS3 with final O_2_ levels <1 μM (indicating severe hypoxic conditions). The fall in O_2_ levels upon submergence with either washing or loading solution was less rigorous (reaching final O_2_ levels of ~18 μM and ~36 μM, respectively).

### Changes of malondialdehyde and soluble sugars during cryopreservation

**Fig. 4:**
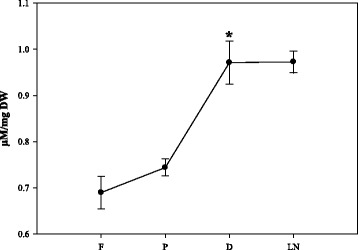
Malondialdehyde (MDA) accumulation during cryopreservation. Cryo-steps are indicated along the x-axis (F- fresh; P – preculture; D – dehydration; LN – Storage and recovery). Data represent the mean ± SE (*n* = 5). *p < 0.05 using a paired Student’s t test

**Fig. 5:**
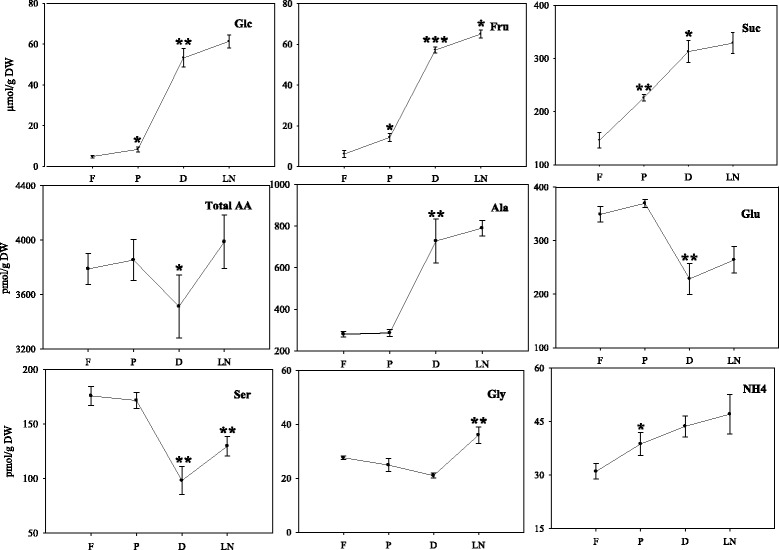
Determination of soluble sugars (Glc – Glucose; Fru – Fructose; Suc – Sucrose), amino acids (Total AA – Total amino acid; Ala – Alanine; Glu – Glutamic acid; Ser – Serine; Gly – Glycine) and ammonium (NH_4_^+^) during cryopreservation. Cryo-steps are indicated along the x-axis (F- fresh; P – preculture; D – dehydration; LN – Storage and recovery). Data represent the mean ± SE (*n* = 5). *p < 0.05. **p < 0.01, ***p < 0.001 using a paired Student’s t test

Quantification of MDA revealed that up to the dehydration step, an increasing concentration of MDA was detected and almost no further increase was observed after LN storage (Fig. 4). MDA accumulation was higher after dehydration (*P* < 0.05, paired Student’s *t*-test) in comparison to the preculture step. The levels of glucose, fructose and sucrose showed similar patterns during the cryo-procedure with large increases (*P* < 0.05, paired Student’s *t*-test) in the dehydration step (Fig. 5).

### Alteration of soluble amino acids during cryopreservation

The total amount of amino acids did not show any remarkable changes throughout the whole cryo-procedure (Fig. 5), however they were slightly lower (*P* < 0.05, paired Student’s *t*-test) at the dehydration step. Interestingly, 2.5 fold of alanine was increased during the dehydration step and it became the major amino acid in dehydrated garlic tips. Total amino acids as well as alanine and glutamic acid showed no significant change in response to LN storage whereas glycine and serine (*P* < 0.05, paired Student’s *t*-test) were increased after LN storage. Throughout the cryo-procedure, the level of GABA was unchanged (data not shown). In addition, the concentration of ammonium increased in the cryopreserved explants compared to the fresh ones whereas preculture step showed a notable increase (*P* < 0.05, paired Student’s *t*-test).

**Fig. 6:**
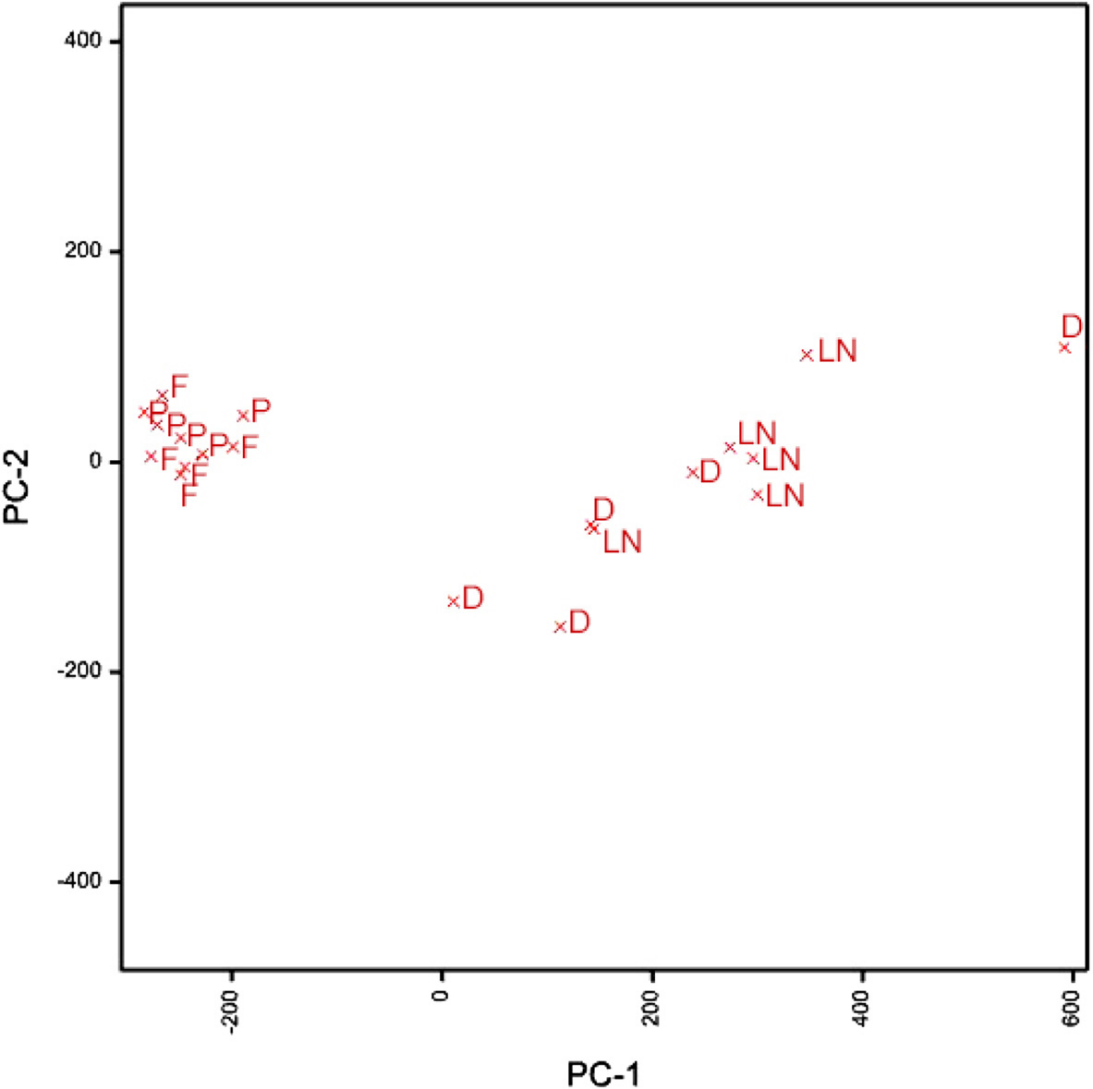
PCA analysis of the most responsive amino acid profiles such as alanine, glutamic acid, glycine and serine, which represent shoot apices in the course of cryopreservation. Samples were projected into bi-plots of principal components that were arranged in descending order of variance. Each of the first five principal components allowed clear distinction of metabolite profiles from samples of cryo-steps F- fresh; P – preculture; D – dehydration; LN – Storage and recovery)

A Principle Component Analysis (PCA) displayed amino acid profiles of alanine, glutamatic acid, serine and glycine in a scores plot (Fig. 6) and revealed that PC1 significantly separated cryo-steps into unstressed (fresh and preculture) and stressed (dehydration and LN storage) conditions. In this plot the changes during dehydration and LN storage respectively did not cluster. Obviously, changes did not appear in fresh and precultured explants either.

Overall, the differences in amino acid accumulation in cryopreserved explants lay with the accumulation of alanine under hypoxia and glycine with cold stress in the dehydration and LN storage, respectively.

## Discussion

Plant shoot apices do not survive exposure to liquid nitrogen without cryoprotective treatments [18]. The vitrification-based cryopreservation improves survival of plant material by increasing cell sap viscosity and preventing formation of harmful intracellular ice crystals, but it also produces complex stresses [19]. In general, the success rate of cryopreservation is mainly depending on the resistance of explants towards abiotic stresses underlying cryopreservation. Dehydration of samples with PVS2-based vitrification induces mainly chemical cytotoxicity (DMSO, Ethylene glycol) and additionally osmotic stress (glycerol, sucrose), while PVS3 induces osmotic stress [20].

**Fig. 7:**
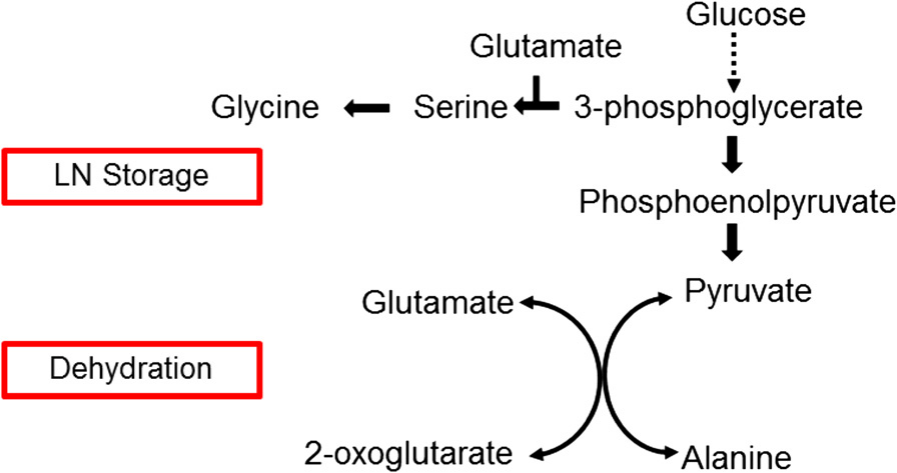
The scheme represents the effect of cryo-stress on amino acid metabolism during dehydration and LN storage steps. Hypoxia led to the redirection of amino acid metabolism, particularly towards accumulation of alanine and decrease of glutamic acid synthesis. Independent of the dehydration stress, LN storage is linked to the accumulation of serine and glycine

Our study demonstrates for the first time that the PVS3-based dehydration also induces severe O_2_ deficiency inside the garlic apex, which is accompanied by characteristic changes in amino acid metabolism (see also scheme in Fig. 7). Hypoxia is induced by two major aspects: (1) the PVS3 solution used at the dehydration step has rather low oxygen solubility, and (2) it additionally hinders gaseous diffusion by its viscosity. Consequently, the immersion of garlic apices leads to strong restrictions in oxygen diffusion into the tissue, followed by rapid decline in the internal O_2_ levels. Immersion of tissues in PVS3 appears to have an effect similar to that of waterlogging, which typically induces hypoxia/anoxia in roots/rhizomes. Waterlogging hampers oxygen diffusion (approximately 10,000 times slower than in water than in air) and ultimately diminishes the flux of O_2_ [21, 22]. The region in and adjacently around the garlic meristem was found to be hypoxic (Fig. 1). The foliage leaf bases around the meristem dome were initially observed to be well oxygenated (~95 μM O_2_), but immersion in PVS3 solution during dehydration caused sudden, dramatic decline in O_2_ level around the foliage leaf bases (~0.85 μM) (Fig. 3). This reduction was also observed in other cryopreservation solutions such as loading (36.4 μM) and washing (18.2 μM) solutions. Low oxygen supply to meristematic tissues might have several implications for plant performance and probably also for plant survival. Greve *et al.* [23] found that shoot meristems were more vulnerable to anoxia than roots and rhizomes. In account with this result we found lower survival rates of shoot apices after dehydration (Fig. 2). The limited availability of oxygen has been reported to have many effects on plant metabolism and physiology, negatively affecting growth and productivity of economically important species [24]. Whereas there were many reports of hypoxia under natural conditions [11] and experimental in vitro conditions [10], this is the first report on the induction of hypoxia in plant tissues undergoing cryopreservation.

Alterations in alanine and glutamate metabolism in garlic apices observed in this study are probably related to the induction of O_2_ deficiency. In previous studies alanine was shown to be the characteristic amino acid accumulated when plants *Arabidopsis thaliana* [25] or *Hordeum vulgare* [26] were suffering from hypoxia. It has been suggested that alanine acts as a non-toxic form of carbon and nitrogen storage during hypoxia, since it firstly allows glycolysis to run under severe O_2_ limitation, secondly it is easily reconverted back to pyruvate (Fig. 7) and/or participates in the synthesis of other amino acids during the recovery period when returning to normoxia [25, 27], and finally its synthesis does not induce shifts in pH. We have demonstrated here that the increase in alanine levels (i.e. alanine synthesis) occurs mainly readily during the dehydration step, which obviously induces hypoxia inside the shoot apex. The accumulation of alanine represents a marker for hypoxia, and alanine synthesis can be used for the diagnosis of hypoxia-related injuries [26]. The relationship concluded here between alanine accumulation and hypoxia in garlic can quite possibly be extended to other plant species in which cryopreservation is being practiced.

After dehydration, alanine became the predominant amino acid (21 %). Similar results were reported by Limami *et al.* [12], who detected that after hypoxic stress alanine replaced asparagine as the predominant amino acid. Moreover, glutamate concentration decreased during the hypoxic dehydration step, which is consistent with its role as precursor of alanine synthesis [12].

MDA is a marker of peroxidation caused by reactive oxygen species (ROS). In the present study MDA content increased significantly in the preculture, reached its maximum during dehydration and slightly increased after LN storage (Fig. 4) indicating that the generation of ROS is highest after the hypoxic dehydration step. This finding is consistent with other cryopreservation studies [28]. The presented results show that the increase of MDA is associated with a decrease of the regeneration potential. This corresponds to the observations of Verleysen et al. [29] indicating that MDA is a parameter for loss of viability due to cold stress and chilling injury. This is reported to have accumulated in plant tissues under hypoxic conditions [30] and MDA was enhanced maximally of 1.4 fold during dehydration. Altogether, the cryo-stress obviously had deleterious effects on the shoot apices, because the survival rate determined after cryopreservation was much lower (47.1 %) and the explants grow slower than without these influences (Fig. 2). Apart from the stress markers, soluble sugars have important roles in different protection systems, such as in the reactive oxygen species balance [31]. An increasing pattern was observed in soluble sugars throughout the cryoprevation protocol (Fig. 5). Indeed, sucrose is known to be a very important metabolite that is involved in different pathways and has been observed to be a key molecule in determining the ability of plants to be cryopreserved [32, 33]. Furthermore, sucrose plays an important role as cryoprotectant [34]. The tissue level sucrose enhancement across cryo-steps might also be related to the increased supplementation of sucrose concentration in cryopreservation solutions.

An increase in total amino acid content has already been reported for many plant species submitted to O_2_ deficiency [35]. In the present study this was not observed during the dehydration step (Fig. 5), which might be due to the short incubation time of only two hours. As vitrification avoids ice crystal formation and protects plant cells from freezing, this process could not be considered as proper cold stress. But vitrification may have features in common with cold stress. The concentration of the amino acids glycine and serine increased after LN storage which resembles changes occurring after cold stress [36]. Serine has been demonstrated to accumulate in *Lolium perenne* at low temperature conditions [37].

As serine is closely linked to glycine formation [38], the concurrent accumulation of glycine was probably measured after LN storage. Furthermore glycine accumulation at this step can be related to a change in lipid peroxidation indicated by a stagnation of the MDA concentration [39].

## Conclusions

This study analyzed the stress factors involved in the different steps of vitrification and documented the occurrence of hypoxia in addition to osmotic stress during dehydration. This might be of high relevance in regard to the reduced regeneration rates after cryopreservation and could lead to improvement of the protocols. Understanding the mechanisms of cryo-stress will also help us to understand how plants respond to various types of abiotic stresses in nature. Further studies are necessary that examine hypoxia during dehydration at molecular level.
